# New Ideas

**Published:** 1856-12

**Authors:** 


### NEW IDEAS.

MOURNFULLY pleasing are many of the reminiscences of our childhood. EBENEZER SHARPE, so portly, rubicund, and good! He inaugurated us into our Latinity. But of all he said and did, an act and a fact only now remain—a theory and a practice. Of the two, we rather think the practice was the more indelible. It was a practice, too, of singular uniformity and regularity, to give the girls a kiss of a morning. There was one, the sweetest of them all, whom, in forgetfulness, he would kiss twice. The theory, rather less impressive, but as durable, was in these words:

"Remember, young gentlemen, every new idea is worth a silver dollar to you."

According then, to this money value of ideas, we find ourselves enriched to-day, and own our indebtedness to Wm. G. Haeselbarth, Esq., the editor of the *Rockland County Journal*, published at Nyack, N. Y., to the reading matter of which paper do we feel our indebtedness from time to time. Says our confrere, "If health is a duty, to subscribe for *Hall's Journal of Health* must be a duty also; for by means of one, the other can be preserved." Q. E. D.

